# Phytolacca Decandra, Modus Operandi and Therapeutic Value Of; with a Case of Poisoning by the Tincture of the Root

**Published:** 1866-07

**Authors:** A. W. Griggs

**Affiliations:** West Point, Georgia


### ORIGINAL COMMUNICATIONS.

### ARTICLE I.

*Phytolacca Decandra, Modus Operandi and Therapeutic Value of; with a Case of Poisoning by the Tincture of the Root.* By A. W. Griggs, M. D., West Point, Georgia.

The use of the root and berries of the phytolacca decandra, as a domestic remedy, and reputed therapeutic value of the same, vouched for by many medical gentlemen of standing in the profession, (and also by those not altogether orthodox,) induce me to resume the pen, and venture once more before your readers. You will find in perusing the following report, that a certain train of symptoms was observed, more like those produced by nux vomica and its preparations than any that have yet been described, as resulting from the use of the poke. This will of course be a point of interest to the therapeutist.

Without further preliminaries, allow me to state, that on the morning of the nineteenth of February last, at eight o'clock, I visited the family of Mr. Wilson, nearly a mile distant, and was invited to examine and prescribe for his child—a male mute of six years old. Was informed that an hour or so previously he had swallowed a small quantity (fluid drachms two or three,) of the tincture of the root of the common poke. His extremities were stiff; hands firmly shut; feet extended and toes flexed; eyes bleared and dancing, pupils contracted, inferior lids drawn down; teeth clenched; lips everted and firm: muscular rigidity was general, and episthotonos established. The circulation numbered eighty-five beats per minute; pulse soft and unresisting; temperature nearly natural; the respiration difficult and oppressed; mucus rale distinctly audible anywhere in the room. The contraction of the *masseters* precluded the idea of addressing remedies per orem, and the amount of mucus in the bronchiæ that of administering anæsthetics. A boy was dispatched to the office for cupping case and mustard; returned at nine o'clock. During the hour I had noticed increased muscular rigidity generally, with convulsive action of the muscles of the face and neck, (the chin drawn closely down to the sternum); which condition would last five or ten minutes, to be succeeded by partial relaxation, and return in twenty minutes more, with the same violence. Cold had been applied to the scalp, and I now proceeded to cup him freely over the temples, the cervical and dorsal spine, and subsequently applied a sinapism from atlas to sacrum. A stream of cold water was now gently poured upon the head, almost constantly for an hour; when the symptoms began to abate, the muscles gradually losing the *tetanic* spasm, and complaint was made of the mustard. At noon he was able to drink a cup of sweet milk which was given. He then slept twenty or twenty-five minutes, (which he had not done before,) and was awakened by slight jerking and twitching of the muscles, especially those of the inferior extremities. He also made signs indicating pain in the back of the head and in the stomach. The water was re-applied, and another cup of milk given. The head was then enveloped by wet towels, which were frequently changed, and the milk allowed ad libitum. At four o'clock he again fell asleep, and rested quietly for an hour and a half. The distressing symptoms had now almost entirely disappeared, and I left the patient, having first ordered a dose of castor oil to be given at bedtime. On the following day the little boy was up and frolicsome, although sore and somewhat stiffened. Nothing more was prescribed, and he recovered without a return of any of the previously mentioned symptoms, and there was no sequela. In conclusion, I think it proper to state that there was retention of urine for ten hours, and that neither emesis nor catharsis took place in this case until the latter was prompted by the use of castor oil; further, the slightest nausea was not at any time observable.

Having positive proof that the patient had taken the drug, and nothing else—not even breakfast—I was at first perplexed knowing that such symptoms in general had not been, before, ascribed to the use of the phytolacca. [See U. S. Dispensatory.] Now was this an idiosyncratic case? Ground is here left for experiment and speculation. From the views which I have long entertained as to the pathology of rheumatism, and the known efficacy of the phytolacca in controlling it, after the acute stage has passed, I am greatly inclined to believe that the symptoms referred to in the case described were such as might have reasonably been expected to have occurred. I am free to admit that we have for some time been at sea respecting the nature of rheumatism; and I cannot, in this article, do more than allude to the fact that, agreeably to my experience, that plan of treatment is most successful which is based upon the idea or belief that the *medulla spinalis* is at fault. Therapeutics often dissipates the darkness of pathology, although the latter, when understood, invariably should direct the former. Thus you may understand, that if the case here reported might be taken for a criterion so far as the specific action of phytolacca is concerned, the pathology of certain forms of rheumatism would be clear. I am apprised that caution should mark every step in science—that we should not build on an insecure foundation—and as before mentioned, that there is here room for experiment and speculation; but, impressed by the action of phytolacca in this case, and knowing its value in treating rheumatism, and believing rheumatism for the most part due to disorder of the cerebro spinal system, I cannot refrain from looking further on, to inquire whether the tincture of the root of phytolacca decandra may not some day be to strychnia what the tincture of veratrum viride is now to tartarized antimony.

